# Foetal cortical expansion is associated with neurodevelopmental outcome at 2-years in congenital heart disease: a longitudinal follow-up study

**DOI:** 10.1016/j.ebiom.2025.105679

**Published:** 2025-03-29

**Authors:** Siân Wilson, Hyuk Jin Yun, Anjali Sadhwani, Henry A. Feldman, Seungyoon Jeong, Nicholas Hart, Kaysi Herrera Pujols, Jane W. Newburger, P. Ellen Grant, Caitlin K. Rollins, Kiho Im

**Affiliations:** a Fetal-Neonatal Neuroimaging & Developmental Science Center, Boston Children's Hospital, Boston, MA, 02115, USA; b Division of Newborn Medicine, Boston Children's Hospital, Boston, MA, 02115, USA; c Department of Pediatrics, Harvard Medical School, Boston, MA, 02115, USA; d Department of Psychiatry and Behavioral Sciences, Boston Children's Hospital, Boston, MA, 02115, USA; e Department of Psychiatry, Harvard Medical School, Boston, MA, 02115, USA; f Biostatistics and Research Design Center, Boston Children's Hospital, Boston, MA, 02115, USA; g Department of Neurology, Boston Children's Hospital, Boston, MA, 02115, USA; h Department of Cardiology, Boston Children's Hospital, Boston, MA, 02115, USA; i Department of Radiology, Boston Children's Hospital, Boston, MA, 02115, USA

**Keywords:** Foetal, MRI, Congenital heart disease, Neurodevelopment, Longitudinal, Cortex

## Abstract

**Background:**

In adolescents and adults with complex congenital heart disease (CHD), abnormal cortical folding is a putative predictor of poor neurodevelopmental outcome. However, it is unknown when this relationship first emerges. We test the hypothesis that it begins in utero, when the brain starts to gyrify and folding patterns first become established.

**Methods:**

We carried out a prospective, longitudinal case-control study, acquiring foetal MRIs at two timepoints in utero, (Scan 1 = 20–30 Gestational Weeks (GW) and Scan 2 = 30–39 GW), then followed up participants at two years of age to assess neurodevelopmental outcomes. We used normative modelling to chart growth trajectories of surface features across 60 cortical regions in a control population (n = 157), then quantified the deviance of each foetus with CHD (n = 135) and explored the association with neurodevelopmental outcomes at two years of age.

**Findings:**

Differences in cortical development between CHD and Control foetuses only emerged after 30 GW, and lower regional cortical surface area growth was correlated with poorer neurodevelopmental outcomes at two years of age in the CHD group.

**Interpretation:**

This work highlights the third trimester specifically as a critical period in brain development for foetuses with CHD, where the reduced surface area expansion in specific cortical regions becomes consequential in later life, and predictive of neurodevelopmental outcome in toddlerhood.

**Funding:**

This research was supported by the 10.13039/100000065NINDS (R01NS114087, K23NS101120) and 10.13039/100000070NIBIB (R01EB031170) of the NIH, PHN Scholar Award, AAN Clinical Research Training Fellowship, BBRF Young Investigator Awards, and the Farb Family Fund.

**Research in context**

**Evidence before this study**

We evaluated PubMed publications in November 2024, including terms associated with early life imaging such as ‘MRI’, ‘foetal’, ‘neonatal’, ‘prenatal’, ‘postnatal’, and ‘Congenital Heart Disease’, as well as ‘outcome’, ‘neurodevelopment’, and ‘Bayley’. No language restrictions were applied.

**Added value of this study**

With longitudinal MRI data, we were able to track foetuses along a growth trajectory, to more robustly highlight when, and in which cortical areas foetuses with CHD deviate from the norm. In following up the participants after birth, we also provide empirical evidence for an association between foetal cortical structure and motor, language and cognitive skills at 2 years of age.

**Implications of all the available evidence**

We demonstrate an application of normative modelling to sensitively detect aberrant cortical development in a highly heterogenous clinical group when these abnormalities first emerge at the start of life. We overcome the challenges to quantifying and interpreting foetal brain MRI features with spatial and temporal specificity, highlighting their value as a predictive tool for the characterisation of patient groups and identification of vulnerable individuals prior to birth.

## Introduction

Congenital heart disease (CHD) encompasses a range of structural cardiovascular defects present at birth. It is the most common birth defect with an estimated prevalence of 1 in 100 births worldwide.^1^ Clinical care has significantly improved for this patient population in recent decades, including surgical interventions that increase survival into adulthood and overall life expectancy.^2^^,^^3^ However, neurodevelopmental deficits rank among the most common comorbidities experienced by individuals with CHD,^4^, ^5^, ^6^, ^7^, ^8^, ^9^ including developmental delays, learning disabilities, and challenges to living independently.^3^ One major contributor to these impairments is thought to be altered prenatal brain development, however few studies have explored this hypothesis longitudinally, tracking the same individuals from prenatal to postnatal development.

The advent of foetal brain MRI has highlighted aberrant brain development in foetuses diagnosed with CHD before birth. Foetuses with CHD have smaller subplate, subcortical and overall cerebral volumes,^10^, ^11^, ^12^, ^13^, ^14^, ^15^, ^16^, ^17^ reduced cortical grey matter volume,^18^, ^19^, ^20^ and altered brain morphometry.^21^ These findings shifted the focus towards antenatal and preoperative factors driving altered brain development in CHD, such as altered foetal haemodynamics,^12^^,^^15^^,^^16^^,^^21^ genetic variants,^22^, ^23^, ^24^ placental function,^25^, ^26^, ^27^, ^28^ maternal stress,^29^ and socioeconomic and environmental factors.^30^ Absent from the literature until recently was follow up of foetuses in later life, to see if abnormal prenatal brain structure is consequential for future neurodevelopment. Recent work from our group established an association between reduced regional brain volumes in foetuses with CHD and worse neurodevelopmental outcomes at two years of age,^30^ suggesting that foetal structural brain MRI metrics could potentially identify vulnerable individuals prenatally. In this study, we extend existing knowledge to investigate whether differences in regional cortical surface morphology are also related to neurodevelopmental outcomes.

Cortical surface area expansion and gyrification are critical developmental processes in the foetal period for brain function and higher-order cognitive ability later in life. Abnormal cortical folding is a putative predictor of poor outcome across many psychiatric disorders,^31^^,^^32^ however, due to the challenges of resolving the foetal cortex with MRI (e.g., motion artefacts, partial volume averaging, and time intensive segmentation), there are limited in utero studies characterising cortical surface maturation in the CHD population. Our previous work described alterations in foetal sulcal patterning in CHD as early as 22 GW,^33^ but found no differences in cortical gyrification index, mean curvature, or surface area compared to the typically developing population. Another study found reduced gyrification in a small sample of 18 foetuses with Hypoplastic Left Heart Syndrome (HLHS).^18^ However, in these smaller cross-sectional cohorts (n = 18–19 subjects) spanning narrow gestational age (GA) ranges (21–30 GW), it is challenging to study the unique growth trajectories of individuals in the heterogenous CHD population, or to compare the effect of different cardiovascular physiologies on cortical morphology. In addition, to the best of our knowledge, there are no previous studies in either CHD or the typically developing population linking abnormal cortical maturation in utero to postnatal neurodevelopment.

To address this knowledge gap, we carried out a prospective, longitudinal case-control study, acquiring brain MRI of typically developing foetuses and foetuses diagnosed with CHD between 2014 and 2023 at Boston Children's Hospital, then following up the infants at 2-years old to carry out neurodevelopmental assessment. We used normative modelling to characterise the maturational trajectory of regional cortical surface metrics in a large cohort of foetuses with CHD relative to a control population. Normative modelling leverages healthy control data to provide a comprehensive reference for ‘normal’ variability across a population, allowing sensitive assessment of an individual's position on a growth curve.^34^ In this work, we normatively model cortical maturation in 60 cortical regions, characterising different aspects of cortical morphology using surface area, sulcal depth, and mean curvature metrics. Leveraging the longitudinal data, we compared the deviance of each foetus with CHD from the norm at two time points on each regional growth curve, the first between 20 and 30 GW, and the second between 30 and 39 GW. We anticipated that aberrant cortical development in the CHD group would only be detectable after the fast growth phase of gyrification, which begins around 28–30 GW for most cortical regions.^35^^,^^36^ With this approach, we tested two main hypotheses: (i) aberrant cortical growth in foetuses with CHD emerges in the late third trimester, confined to specific cortical regions; and varies according to the type of cardiovascular impairment; (ii) greater deviance from the normative growth curves is associated with lower neurodevelopmental outcomes at two years of age.

## Methods

### Ethics

Ethical approval was granted by the Boston Children's Hospital Institutional Review Board (IRB approval number: IRB-P00008836), and written, informed consent was obtained from mothers prior to participation.

### Participants

From 2014 to 2023, we performed a prospective, longitudinal cohort study.^15^^,^^30^ The inclusion criteria for CHD participants was pregnant women with a foetal diagnosis of ‘moderate to severe CHD’, according to the categorisation by Hoffman and Kaplan.^30^^,^^37^ Our exclusion criteria were maternal age <18 and >45 years, multiple gestation, maternal CHD diagnosis, foetal genetic or extracardiac anomaly detected with clinical testing, brain malformation, and congenital infection. The Control population included some foetuses with a family history of CHD (n = 60), but who had a normal foetal echocardiogram and no neurological concerns.

The final study cohort included 216 participants, of which 140 were scanned once (CHD = 47, Controls = 93) and 76 were scanned twice (CHD = 44, Controls = 32), the GA at scan for MRI 1 was between 20 and 30 GW and MRI 2 at 30–39 GW. The mean interscan interval was 8.33 weeks (interquartile range; 5.86–10.04 weeks). Overall, the study included 292 in utero 3T T2-weighted brain MRI scans (20–39 GW) (CHD = 135, Controls = 157), We collected demographic information as shown in Table 1, including sex (assigned by a clinician at birth), GA, maternal age, maternal education, self-reported maternal race and ethnicity, and birth metrics (e.g., GA at birth, birth weight). For the CHD group, we also report their diagnosis and cardiovascular physiology, which was extracted from the clinical echocardiogram.

**Table 1:** Cohort demographic information.

Foetal characteristics	CHD	Controls
Total number of scans	n = 135 (subjects n = 91)	n = 157 (subjects n = 125)
Sex (male subjects)	n = 58 (64%)	n = 59 (47%)
Gestational age (GA) (weeks)	19.6–38.8 (*median* = 30.7)	22.0–38.7 (*median* = 31.3)
Longitudinal subjects (two in utero scans)	n = 44 (48%)	n = 32 (26%)
Scan 1 GA (weeks)	19.6–30.4 (*median* = 28.4)	22.0–30.0 (median = 27.6)
Scan 2 GA (weeks)	30.0–38.80 (*median* = 36.4)	29.1–38.7 (*median* = 36.0)
ΔScan (median, range)	(7.4, 3.7–16.6)	(7.5, 3.3–14.4)
**Postnatal characteristics**		
GA at birth (weeks)	32.4–40.6 (*median* = 38.8)	34.6–41.9 (*median* = 39.2)
Birthweight (g)	1675–4880 (*median* = 3242)	2297–4200 (*median* = 3481)
Bayley III follow-up	n = 51	n = 38
Mean age at assessment (months)	23.7	23.2
**Maternal characteristics**		
Maternal age at scan (years)	31.3 ± 4.5	32.6 ± 4.3
Maternal education, college or higher	n = 31	n = 26
**Maternal race/ethnicity**		
White	n = 65	n = 96
Black/African American	n = 1	n = 5
Asian	n = 3	n = 3
Hispanic ethnicity	n = 10	n = 11
Other/unknown	n = 12	n = 10
**Cardiac factors**		
HLHS/TGA cases	Subject n = 39 (43%) (scan n = 63)	–
Cardiac catheterisation	n = 14 (15%)	–
Cardiac surgery	n = 27 (30%)	–

### T2-weighted brain MRI acquisition & processing

Pregnant women were scanned on a 3-T Siemens MRI scanner in supine position. We acquired multi-planar repeated T2-weighted Half-Fourier Acquisition Single-Shot Turbo Spin-Echo (T2wHASTE) sequences with a 2 or 4 interleaved acquisition; TE = 100–120 ms, TR = 1400–2000 ms, slice thickness = 2–3 mm, variable field of view dependent on foetal and maternal size, 256 × 204, 256 × 256, or 320 × 320 acquisition matrices and in-plane resolution of 1 mm.^15^^,^^38^ Total acquisition time was approximately 30 min.

**Fig. 1:**
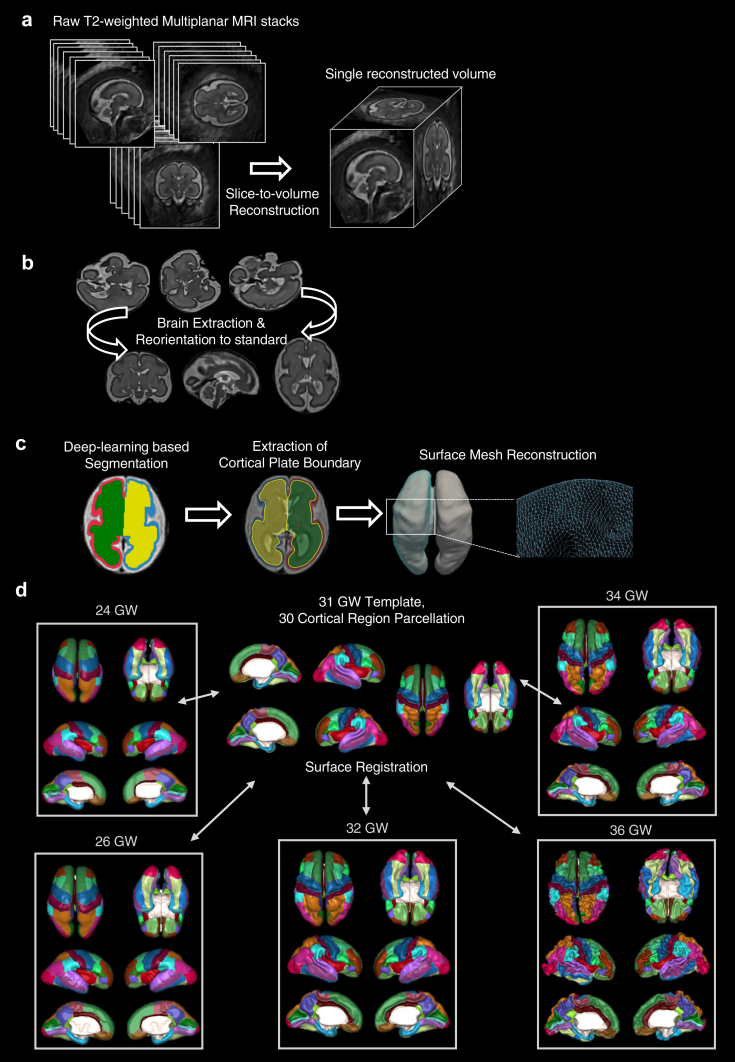
**Summarised workflow of the image processing pipeline. (a)** In utero multiplanar MRI stacks are acquired, then slice-to-volume reconstruction is used. **(b)** Brain extraction is performed to remove the surrounding maternal tissues and volumes are reoriented to standard space (spatial normalisation step). **(c)** A deep learning network is used for foetal brain tissue segmentation, to estimate the cortical plate. The inner boundary of the cortical plate is then extracted and used to reconstruct the surface mesh. **(d)** Each individual subject surface is registered to a 31 GW surface template, which is used to delineate 30 cortical regions in each hemisphere. The cortical parcellation is propagated from the template back to each individual subject surface to conduct subsequent regional analysis. Examples of the foetal cortical labels propagated to individual subject space at different gestational ages are shown.

The image processing steps are briefly summarised in Fig. 1. Patients were deidentified during the image reconstruction process and MRI volumes were given a serial number. MRI volumes were reconstructed and segmented with our bespoke in-house pipeline.^35^^,^^39^ We first used a foetal brain extraction model, based on a 2D U-net structure, trained with 291 MRI stacks from 65 typically developing foetuses (https://github.com/FNNDSC/foetal-brain-segmentation). We then applied N4 bias field correction to adjust for intensity inhomogeneity^40^ and created a motion-corrected 3D volume with 0.75 mm isotropic resolution using a slice-to-volume super-resolution technique.^41^ To extract the cortical plate (CP), we developed a foetal-specific automated segmentation algorithm (https://github.com/jwhong1125/foetal_CP_segmentation)^42^ based on a 2D U-net model trained separately for the sagittal, coronal, and axial planes. It includes multi-view aggregation and test-time augmentation for precise CP segmentation onto the 3D volumes.

### Inner cortical plate surface reconstruction

Using the inner CP boundary, we reconstructed the surface of left and right hemispheres independently, by applying the marching-cube algorithm in the CIVET-2.1.0 package (https://mcin.ca/technology/civet/).^43^ Inner parts of the segmented CP were binarised and the initial meshes were tessellated by fitting the boundary of the inner part of CP. We resampled the initial meshes to the standard format surfaces containing 81,920 triangles and 40,962 vertices.^43^ To eliminate geometric noise, Taubin smoothing was applied to the surfaces,^44^ to obtain a natural shape of the surface without shrinking and voxelated patterns. We then visually checked the quality of the surface reconstructions by overlaying them onto the original T2 and the CP segmentation using Freesurfer (https://surfer.nmr.mgh.harvard.edu/).^45^

### Surface registration and parcellation

We registered individual cortical surfaces to the 31 GW template surface^35^^,^^46^^,^^47^ using the CIVET-2.1.0 2D sphere-to-sphere warping method,^48^^,^^49^ which searches for optimal correspondence of vertices based on the folding similarity between individual and template surfaces.

To parcellate the cortex, we manually refined the FreeSurfer Desikan adult parcellation^50^ from 34 regions to 30 cortical regions in each hemisphere on our 31 GW foetal surface template, under the guidance of a neuroradiologist (Fig. 1). Since the foetal brain is significantly smaller and the folding of the cortex is not fully mature, certain parcels were merged, including three subdivisions of cingulate merged to one, banks of the superior temporal sulcus were divided into two parts and merged them to superior and middle temporal sulci, entorhinal merged to hippocampal gyrus.

Using surface registration, the 30 foetal labels were propagated from the 31 GW template to each individual subject using the aforementioned warps, and evidence for the success of this approach across the gestational age range is shown in Fig. 1, and in recently published studies from our group.^35^^,^^51^ A visual quality control (QC) to check the alignment of the labels with major sulcal landmarks was performed to determine the final number of foetal surfaces that could be included for the analysis, a flowchart showing the number of subjects excluded at each stage of the image processing pipeline is shown in Supplementary Figure S1.

### Cortical surface metric calculation

Three metrics describing surface features were chosen to capture cortical folding morphology, surface area, sulcal depth, and mean curvature. The surface area was computed according to the Voronoi region around each vertex on the cortical surface.^52^ Sulcal depth was calculated using the adaptive distance transform method.^53^ For the mean curvature, we calculated the angular deviation from a patch around each vertex^52^ and used the absolute mean curvature to measure the complexity of the cortical folding shape. We calculated the surface area, average sulcal depth, and average absolute mean curvature for each of the 30 cortical regions in each hemisphere.

### Gaussian process regression of surface metrics

We used the typically developing control population to normatively model the trend in mean surface metrics across GA for each cortical region. We used Gaussian Process Regression (GPR), a Bayesian nonparametric regression, implemented in GPy (https://sheffieldml.github.io/GPy/). We use a Radial Basis Function (RBF) kernel with input dimension = 1, variance = 1.0, and length scale = 2.0, ensuring smooth function approximation. We used a fixed gaussian noise variance = 0.01, to prevent overfitting. The hyperparameters were initialised at 1.0, with a positive constraint, and optimised over 100 iterations.

**Fig. 2:**
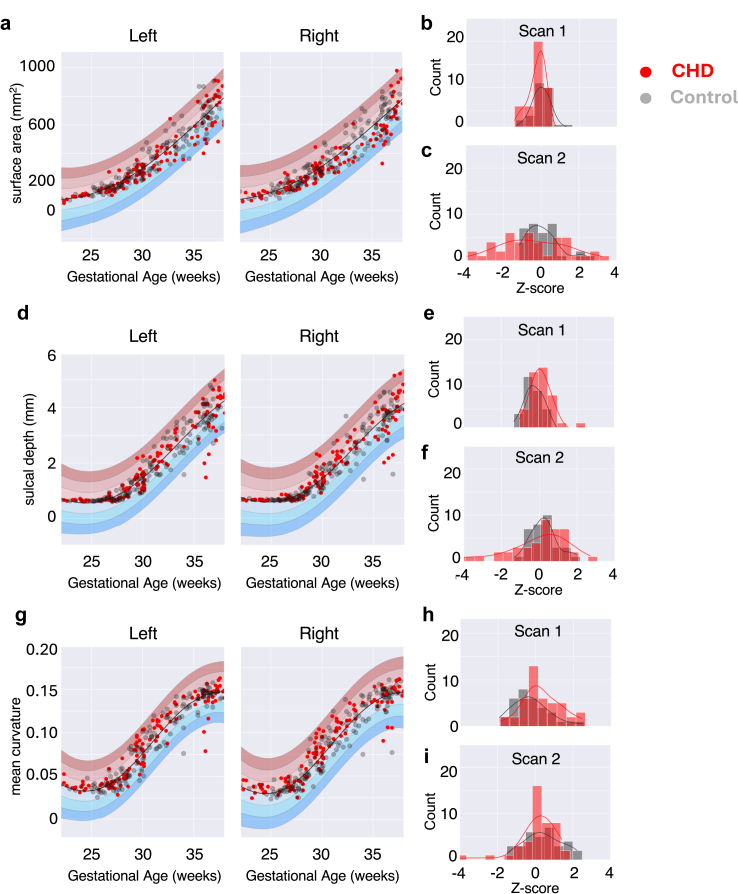
**Normative modelling of surface features across 30 cortical regions**. **(a)** Exemplary cortical region (precentral gyrus) growth chart displaying normative modelling of surface area Control foetuses (n = 157) in grey, CHD (n = 135) in red. Model mean delineated in black, ±1, ±2, and ±3 standard deviations. **(b)** The distribution of z-scores for the first and **(c)** second scans. **(d–f)** Same as above but for sulcal depth. **(g–i)** For mean curvature values.

As detailed in previous studies,^16^^,^^54^^,^^55^ GPR simultaneously provides point estimates and measures of predictive confidence, estimating a continuous standard error of the mean for modelled covariates, in this case GA.^34^ We calculated Z-scores for each scan in each foetus, computing the difference between the predicted and the observed value, normalised by the uncertainty of the prediction (Fig. 2). The Z-scores represent the amount of deviation from the predicted mean for each foetus, in a given surface metric (surface area, sulcal depth, and mean curvature) for a particular cortical region.

### Two-year neurodevelopmental evaluation

Neurodevelopmental assessments were conducted between 18 and 24 months of age using the Bayley Scales of Infant and Toddler Development, Third Edition (Bayley-III),^56^ administered by a licenced clinical psychologist (A.S.). Composite scores (mean ± standard deviation, 100 ± 15) for cognitive, language, and motor skills were computed.

### Statistics

#### Cohort characteristics

To compare group characteristics between CHD and Control subjects (including GA, GA at birth, birth weight, and maternal age) a two-sample t-test was used. The group difference in foetal sex, maternal education and maternal race/ethnicity was assessed using a chi-square test. The results of these statistical comparisons are shown in Supplementary Table S1.

#### Categorising foetal CHD

To explore the impact of severe cardiovascular impairment on the surface features of the foetal brain, we divided the CHD group into two categories, (i) Hypoplastic Left Heart Syndrome (HLHS)/Transposition of the Great Arteries (TGA) and (ii) Other CHD. Detailed diagnoses and the corresponding group of each subject can be found in Supplementary Table S2. This classification has been used previously,^15^ as HLHS and dTGA are diagnoses which substantially reduce substrate delivery to the brain,^57^^,^^58^ in contrast to other lesions that may result in more modest reductions to cerebral oxygen or substrate delivery. In addition, previous work has suggested that brain volumetric differences were greatest in foetuses diagnosed with TGA or HLHS.^15^

#### Group differences in surface metric Z-scores

We performed multiple statistical tests to compare Z-score distributions between groups. We first carried out factorial analysis of variance (ANOVA) to establish group differences between CHD and Control subjects, using the entire cohort and full age range (CHD n = 135, Control n = 157 subjects). Where repeated scans were included for a single subject, the ANOVA included a random effect to account for within-subject correlation. The ANOVA included interaction terms to estimate the group difference (CHD vs. Control) separately for each of the 30 cortical regions. The full equation for the factorial ANOVA can be found in the Supplementary Information. We then confined the analysis to subjects with repeated scans (CHD n = 88, Control n = 64), using ANOVA to compare the CHD Scan 1 vs. Control Scan 1, and CHD Scan 2 vs. Control Scan 2. Then, to boost our statistical power, we included all MRI scans, grouping them by GA into pre-30 GW (<30 n = 143), and post-30 GW (≥30 n = 149), matching the age range of Scan 1 and Scan 2, comparing (i) CHD (<30 GW) vs. Control (<30 GW) and (ii) CHD (≥30 GW) vs. Control (≥30 GW). Z-score differences were calculated between scans, (ΔZ_Scan_ = Z-score Scan 2 – Z-score Scan 1) and compared CHD to Controls, again using factorial ANOVA and adjusting for GA difference between scans (ΔGA = GA Scan 2 – GA Scan 1).

Finally, we subdivided the CHD cohort as described above and used factorial ANOVA to compare (i) CHD (HLHS/TGA) vs. Controls; (ii) CHD (Other) vs. Controls; and (iii) CHD (HLHS/TGA) vs. CHD (Other). We analysed these subgroups in two GA ranges, <30 GW and ≥30 GW, replicating the age range of the longitudinal data. In each ANOVA we used iterative reweighting to identify outliers and reduce the effect of extreme values. To each set of region-specific results, we applied the Holm step-down procedure to correct the 30 p-values for multiple comparisons. We took Holm-adjusted p < 0.05 as the criterion for statistical significance and calculated the false discovery rate (FDR) for the set of significant results thus identified.

#### Multivariate linear regression of surface metric Z-scores and neurodevelopmental outcomes

Stepwise Akaike Information Criterion (AIC)^59^ was used to select the other appropriate covariates for the general linear model (GLM). We applied stepwise AIC selection to test which combination of postnatal, maternal and cardiac factors gave the best model performance in the regression (Bayley III Outcome Score ∼ Z-score + …). The covariates that were assessed included the effect of multiple scans, foetal sex, GA at birth, birth weight, maternal race, ethnicity, education, maternal age, education, and race/ethnicity at delivery, cardiac catheterisation and cardiac surgery (Yes/No). AIC highlighted ‘GA at birth’ and ‘maternal education’ as significant covariates for cognitive, language and motor outcomes, and these variables were therefore included in the regression models, shown below.

The first GLM we fit was to test the hypothesis that Z-score was related to outcome in foetuses with CHD or Controls.

1) Bayley III Outcome Score ∼ Z-score + Group (CHD/Control) + (Z-score ∗ Group) + GA at Birth + Maternal Education

The second GLM included a ‘Pre/Post 30 GW’ term to test whether there was a different relationship between Z-scores before 30 GW (in Scan 2) compared to Z-scores ≥30 GW.

2) Bayley III Outcome Score ∼ Z-score + Group (CHD/Control) + (Z-score ∗ Group) + Pre/Post 30 GW + (Z-score ∗ Pre/Post 30 GW) + GA at Birth + Maternal Education

We used FDR to correct for multiple comparisons across all cortical regions (60) for a given Bayley score.

### Role of funders

The funders had no role in the study design, data collection, data analyses, interpretation, or writing of the manuscript.

## Results

### Group demographics

There were no significant differences in GA at birth, sex, maternal age (two-sample t-test), maternal race or maternal education levels (chi-square test) between CHD and Control Groups (Table 1). For the longitudinal MRI data, the GA range for Scan 1 and Scan 2 was also similar between CHD and Control groups (Table 1).

### Cortical surface area matures abnormally in specific regions, but only in the late third trimester

The normative modelling of surface area, sulcal depth, and mean curvature highlights the rapid expansion and gyrification of the cortex that occurs over the third trimester, with the highest growth rate period beginning around ∼28–30 GW (Fig. 2), as reported in previous work.^35^^,^^36^ We used factorial ANOVA to compare Z-score distributions across the entire cohort, including the full age range, we found no significant group differences between Control and CHD Z-scores for either surface area, sulcal depth or mean curvature.

**Fig. 3:**
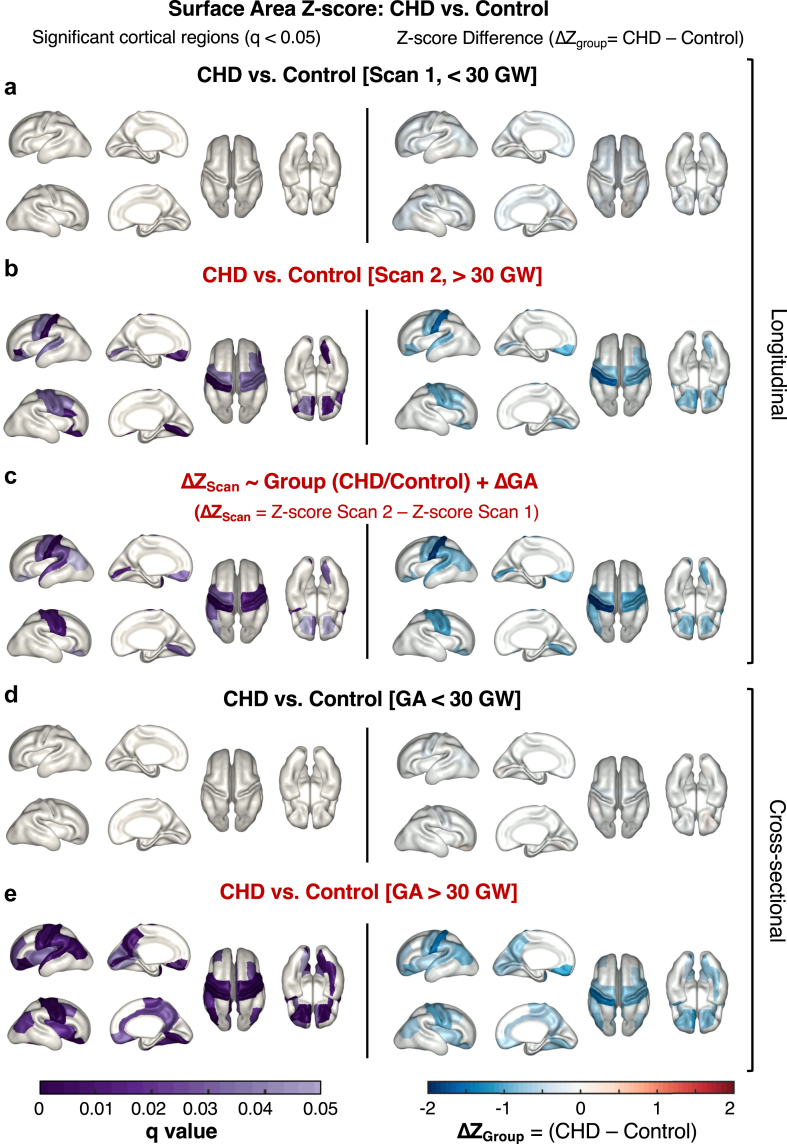
**Cortical regions where surface area Z-score is significantly different between CHD and control foetuses**. **(a)** The factorial ANOVA of Z-scores from Scan 1 showed no significant difference (q > 0.05) between mean Z-scores (ΔZ_Group_) of CHD (n = 44) and Control (n = 32) groups. **(b)** For Scan 2, 13 significant cortical regions and negative ΔZ_Group_, implies reduced surface area in CHD relative to Control population. **(c)** q values (q < 0.05) for 12 cortical regions with significant group differences in (ΔZ_Scan_ = Scan 2 Z-score – Scan 1 Z-score), when accounting for ΔGA (GA Scan 2 – GA Scan 1). **(d)** Repeating the analysis of (a) but including cross-sectional subjects <30 GW (CHD n = 66, Control n = 68), no significant q values (q > 0.05) and no difference between mean Z-scores (ΔZ_Group_) of CHD and Control groups. **(e)** Repeating the analysis of (b) but including all cross-sectional subjects >30 GW (CHD n = 89, Control n = 69).

We then explored only the subjects with sequential foetal brain MRIs, first we examined Scan 1 (19–30 GW) Z-scores in CHD and Controls, again we found no significant differences (Fig. 3a). When then examined Scan 2 (30–39 GW) Z-scores and found significantly different surface area Z-score distributions in 13 gyral regions (q < 0.01), including the pre and post central gyrus, lateral orbitofrontal, lingual, superior temporal and pericalcarine regions (Fig. 3b). We calculated the difference between mean Z-score in CHD and Controls (ΔZ_Group_) for each region and found that the mean Z-score was lower in CHD compared to Controls for all the significant regions (Fig. 3b), indicating reduced surface area expansion in the CHD foetal population for Scan 2. We found only 1 cortical region with a significantly decreased sulcal depth Z-score in CHD and found no differences in mean curvature Z-scores between groups (Supplementary Table S3).

Using the longitudinal data, we explored how each foetus with CHD progressed along the growth trajectory between scans. We again used factorial ANOVA to examine whether the difference between surface area Z-scores at Scan 2 and Scan 1 (ΔZ_Scan_) varied between CHD and Control groups (Fig. 3c). We identified 10 regions with significantly decreased ΔZ_Scan_ in CHD (q < 0.01), that overlapped with the significant cortical regions with decreased surface area from the first analysis (Fig. 3c).

To further explore the finding that foetuses with CHD first deviate from Controls later in gestation, we divided our cross-sectional cohort by gestational age, into a <30 GW (n = 140) and a ≥30 group (n = 152) to boost our statistical power, then carried out our factorial ANOVA on each group separately. We again did not find any significant differences between CHD and Control subjects <30 GW (Fig. 3d), but found 30 cortical regions were significantly different in foetuses ≥30 GW (q < 0.05) (Fig. 3e).

### CHD subtypes are significantly different from controls, but not from each other

**Fig. 4:**
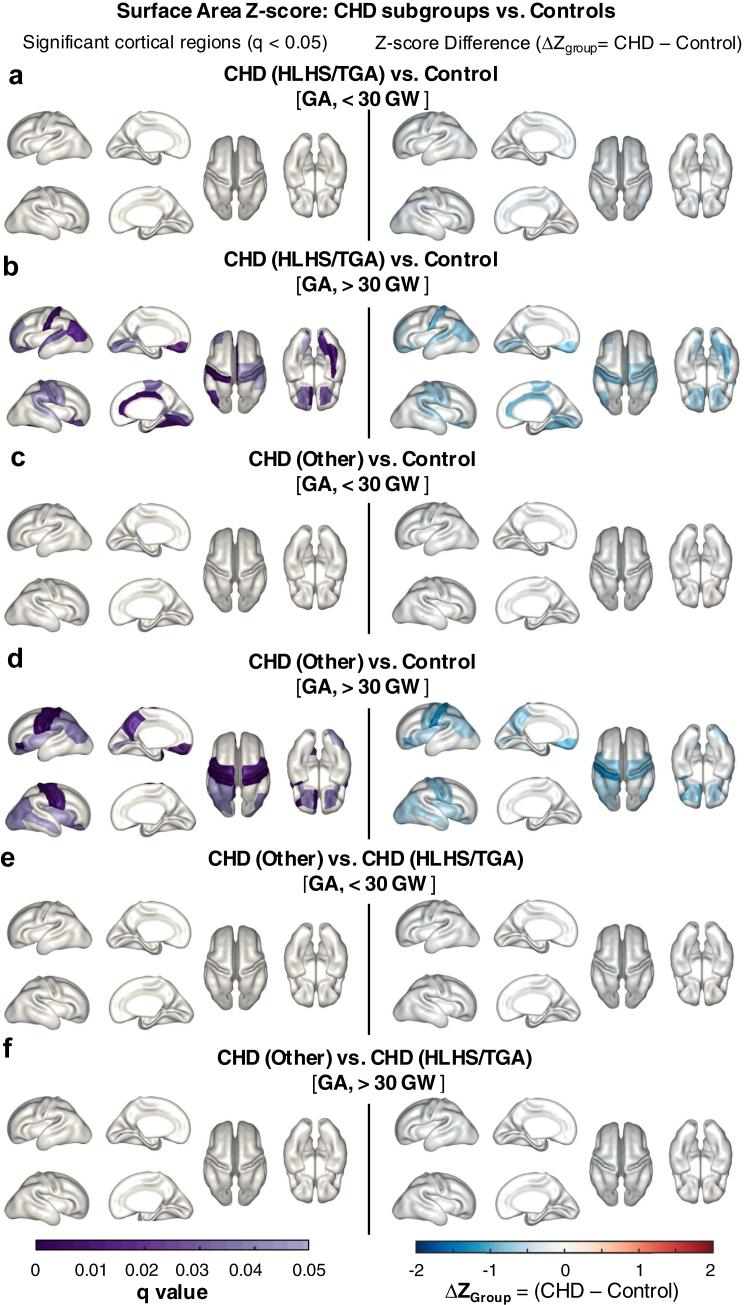
**Surface area Z-score differs between CHD diagnoses**. **(a)** The factorial ANOVA of pre-30 GW showed no significant differences between the CHD (HLHS/TGA) group (n = 32) and Controls (n = 68), (q > 0.05) and no corresponding ΔZ_Group_. **(b)** Post-30 GW there were numerous cortical regions that were significantly different between the CHD (HLHS/TGA) group (n = 31) and Controls (n = 89), after FDR correction applied across 60 cortical regions (q < 0.05). **(c) and (d)** same as (a) and (b) respectively, but for “Other CHD diagnoses” (n = 72) vs. Control foetuses. **(e) and (f)** No significant differences were found between CHD (HLHS/TGA) and CHD (Other) groups for either pre-30 GW or post-30 GW (q > 0.05).

To account for the heterogeneity in CHD, we subdivided the CHD cohort into groups according to features of the cardiovascular physiology, using previously defined categories of (i) “HLHS/TGA” foetuses, diagnoses with theoretically the lowest foetal cerebral substrate delivery; or (ii) “CHD/other”, for all other foetuses with CHD diagnoses.^15^ Again using factorial ANOVA, we tested whether there were differences between these cardiac groups and Controls, grouping them according to pre-30 GW and post-30 GW, and we found no statistically significant difference between cardiac groups, which may reflect a lack of statistical power and need for larger group size. Compared to Controls (Fig. 4), pre-30 GW there were no significant differences in any cortical region (q < 0.05) for either CHD (HLHS/TGA) foetuses vs. Controls (Fig. 4a), or CHD (Other) foetuses vs. Controls. Post-30 GW, 7 cortical regions showed significant differences in surface area z-score between HLHS/TGA physiology and Control foetuses (q < 0.01), and similarly 7 regions for CHD (Other) vs. Control foetuses (q < 0.01). The ΔZ_Group_ relative to controls was more negative for the “Other” group, implying the greatest impairment in surface area expansion for these subtypes.

### Neurodevelopmental outcome scores vary significantly between CHD and controls

Eighty-nine foetuses (CHD = 51, Controls = 38) were followed up as infants at 2 years old, and their language, motor and cognitive skills were assessed using the Bayley Scales of Infant and Toddler Development, 3rd edition (Bayley III).^56^ To explore whether there were expected group differences between CHD and Control toddlers, we performed a two-sample t-test and found significant lower composite scores for the language and motor skills in the CHD group compared to Controls (Table 2).

**Table 2:** CHD subjects have lower neurodevelopmental outcome scores than controls.

Bayley III	CHD vs. control, two-sample t-test			
	CHD (n = 51) (57%)	Control (n = 38) (43%)	Difference (95% CI)	p
Cognitive composite	μ = 99.9	μ = 104.8	−5 (−10.5, 0.5)	0.075 (ns)
≤85	n = 3 (6%)	n = 2 (5%)		
Motor composite	μ = 95.9	μ = 102.6	−6.6 (−10.7,-2.5)	0.002 (∗∗)
≤85	n = 4 (8%)	n = 2 (5%)		
Language composite	μ = 97.7	μ = 107.7	−10.0 (−17.6, −2.4)	0.02 (∗)
≤85	n = 8 (16%)	n = 3 (8%)		

∗*p* < 0.05, ∗∗*p* < 0.01.

### Slower prenatal surface area expansion is predictive of worse Bayley III scores at 2-years old in CHD

**Fig. 5:**
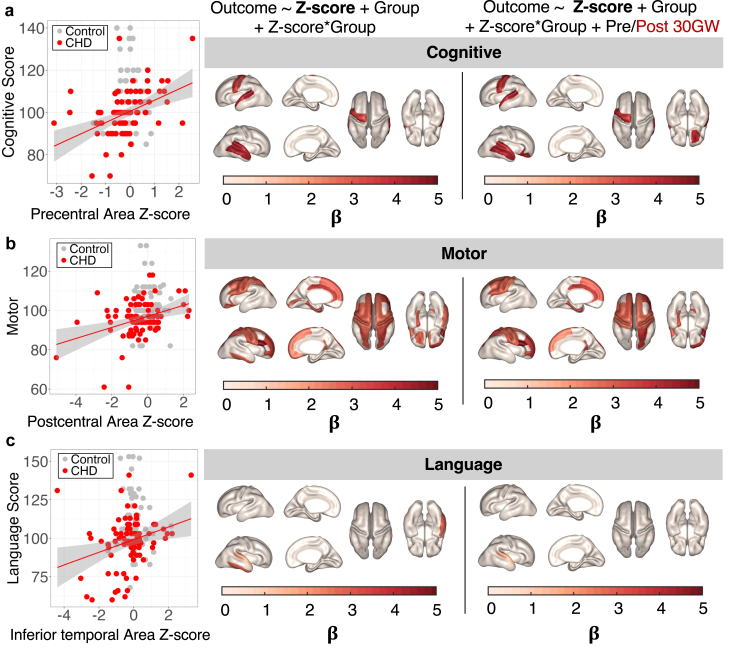
**Multivariate linear regression shows significant correlations between surface area Z-score and neurodevelopmental outcomes in foetuses with CHD**. The beta coefficient for the Z-score term in the general linear model is projected onto the surface for cortical regions where Z-score was a significant predictor of outcome in the general linear model (q < 0.05) for: **(a)** Composite Cognitive score; **(b)** Composite Motor score; **(c)** Composite Language score. Left panel: The relationship between area Z-score and Bayley Scales of Infant and Toddler Development outcome score in CHD (n = 51) (red) and Control subjects (n = 38) (grey) for an exemplary cortical area, demonstrating what the beta coefficients represent. Middle panel: Cortical regions where there was a significant relationship between surface area Z-score and each neurodevelopmental domain. Right panel: Post 30 GW Z-scores are driving the relationship between surface area Z-score and outcome scores.

We fitted GLMs to examine the relationship between surface area Z-scores across all cortical regions and Bayley composite scores (Fig. 5, Supplementary Table S4). For the CHD group, we found multiple cortical regions for which surface area growth was a significant predictor for motor, language, and cognitive composite scores. However, in Controls, we found no significant relationship between Z-score and neurodevelopmental outcomes (Supplementary Tables S4 and S5).

The Beta (β) coefficient describing the relationship between surface area Z-score and outcome measures was projected onto the surface for statistically significant cortical regions (Fig. 5). Six cortical regions, concentrated around the temporal lobe (bilateral superior temporal and transverse temporal gyrus) were positively correlated with the cognitive score (Fig. 5a). We found a significant positive correlation between surface area Z-score and motor composite for 21 brain regions (q < 0.05), this domain also showed the largest β regression coefficients (Fig. 5b). The significant cortical regions included bilateral pre- and post-central, medial frontal, superior frontal, superior parietal gyral areas (Fig. 5b). Language composite score was significantly related to surface area in the inferotemporal, superior temporal and transverse temporal gyrus, lateralised to the right hemisphere (Fig. 5c).

### Z-scores after 30 GW explain variance in outcome measure, but not before

To explore if the Z-scores of older foetuses (≥30 GW) were driving the relationship with outcome, we replicated the study design of the longitudinal cohort and introduced a “pre or post 30 GW” variable to the GLM (Supplementary Table S5). When we included this ‘pre or post 30 GW’ categorical variable, in the CHD group we found a significant effect of surface area Z-scores post-30 GW in 13 brain regions for motor, 2 for language and 5 for cognitive scores (Fig. 5), but no significant effect of pre-30 GW Z-scores. For the control group, there was no significant relationship between the Z-score and any neurodevelopmental outcome for either the pre- or post-30 GW age group (Supplementary Table S5).

## Discussion

The key aims of this study were two-fold, to quantify the emergence of atypical cortical folding morphology in foetuses with CHD, and to understand if aberrant cortical development in utero is associated with neurodevelopmental delays in toddlerhood. To address these aims, we studied a thoroughly characterised, longitudinal sample of foetuses with isolated CHD (no known genetic abnormalities), who underwent neurodevelopmental assessment at 2 years of age. We used normative modelling to characterise the maturation of CHD surface features in utero relative to healthy controls. With this approach we addressed two inherent challenges of studying foetal CHD, 1) the rapid, dynamic development of the foetal brain 2) the subtle, age-dependent differences in brain structure in CHD. This methodology allowed us to highlight the timeframe of onset and regional specificity of deviant surface development in the CHD population in utero. We also found evidence that prenatal deviations from normative surface development correlated with neurodevelopmental outcomes at two years of age. However, a caveat to having a thoroughly characterised cohort is the more limited sample size.

A significant difference in areal expansion between CHD and Control foetuses emerged in 13 cortical regions in the second scan (>30 GW), suggesting that at the macroscale, cortical development in foetuses with CHD follows the expected growth trajectory up until the start of the third trimester (28–29 GW). Leveraging the longitudinal data, we consolidated this finding by showing that ΔZ_Scan_ was also more negative in foetuses with CHD in 12 cortical regions. This result suggests that foetuses with CHD fall below the normative growth curve between first and second MRI scans. Previous work in smaller cohorts also found that differences in cortical growth were only evident after 30 GW.^18^^,^^60^ It is rationalised that this epoch of the foetal period is critical in CHD because of the acceleration in energy-demanding brain growth,^10^^,^^12^ and a corresponding increased complexity of the cortical manifold, facilitating detection of variability between individuals. However, our study showed that sulcal depth and mean curvature followed the expected normative growth trajectory, suggesting that the metabolically demanding mechanisms governing gyrification are largely operational in CHD, despite the developmental strain of abnormal cardiovascular physiology. Other recent work reported similar results, finding no differences in regional sulcal depth in foetuses with CHD, or between CHD subtypes.^17^ Additionally, findings from a neonatal cohort showed that preoperative gyrification at the lobar level is not significantly reduced in CHD when whole brain volume is accounted for.^61^ When interpreted together with the results of this work, this implies that the reduction in cortical folding is due to the smaller overall brain size and reduced cortical expansion of specific regions in CHD. However, the mechanisms driving gyrification may not be significantly affected in CHD pathophysiology.^61^

The deviations we observe in foetuses with CHD were exclusively in surface area expansion, localised to specific brain areas, occurring in the latter half of the third trimester. At the cellular level, the radial unit hypothesis postulates that cortical surface area is driven by the number of ontogenetic columns of neurons, arranged perpendicularly to the surface of the brain.^62^^,^^63^ Our results suggest that specific mechanisms governing the number of neuronal columns are going awry in CHD, in a localised manner, targeted to particular cortical areas. Previous work in animal models has demonstrated targeted effects of altered gene expression on the arealisation and expansion of specific cortical regions, such as the primary visual and somatosensory areas.^64^, ^65^, ^66^ Given the development of distinct cortical areas is influenced by genes that exhibit highly regionalised expression patterns, we therefore rationalise that even in cases of isolated CHD, with no known genetic abnormalities, alterations to specific gene pathways could result in regionally specific alterations to surface area growth. Future work may therefore benefit from a closer, focussed examination of the gene expression pathways driving foetal surface area expansion in the specific cortical regions highlighted by this work.

Prior work on this cohort found the most significant reductions in regional brain volumes in foetuses with HLHS or TGA.^15^^,^^30^ Interestingly, in this work, although we identified no significant differences between the CHD groups, there were more cortical regions that were significantly reduced in CHD (Other) group than the CHD (TGA/HLHS) group (Fig. 4) compared to the Controls. The most significantly different regions between CHD (Other) and Controls were the pre and post central areas, which also had a strong association with outcome in our subsequent analysis. These results suggest that the most abnormal cortical growth was not observed for the diagnoses with theoretically the greatest disruption in prenatal brain oxygen and nutrient delivery (HLHS and TGA). This result suggests that alternative mechanisms other than altered foetal cerebral haemodynamics may be having deleterious effects on foetal brain development and warrant further investigation, such as genetic factors, placental function and the maternal-foetal environment.

The abnormally developing cortical regions in our CHD cohort included the bilateral pre- and post-central areas which are relatively mature at term compared to other regions, both structurally and functionally,^67^, ^68^, ^69^ hence we are able to detect differences in these areas in a foetal cohort. Our analysis also highlighted delayed growth of several frontal lobe regions, including lateral orbitofrontal gyri, medial orbitofrontal, and caudal middle frontal gyrus. This result supports the findings of previous studies in both human and animal models of CHD, in foetal and preoperative neonatal cohorts, identifying abnormal morphometry in frontal cortical areas,^21^ the largest volumetric reduction in the frontal lobe^33^ and reduced surface area in the orbitofrontal and central sulcus regions.^60^ At the microscale, in vivo work in porcine models of CHD reported reduced maturity of astrocytic processes and depleted neuroblasts within the subventricular zone in frontal areas, leading to an excitatory/inhibitory imbalance.^70^ These disruptions at the cellular level may contribute to the abnormal cortical maturation of these frontal brain regions at the macroscale, and the increased risk of developmental delay in CHD.^71^

Previous research in other clinical cohorts has demonstrated that cortical surface area is a strong predictor of neurodevelopmental outcome in childhood.^72^, ^73^, ^74^ However, our study demonstrates the predictive power of prenatal cortical growth as a potential determinant of future neurodevelopment. We demonstrate that the expected differences in motor, cognitive and language development between individuals with CHD and Controls can be explained in part by variations in cortical surface expansion in utero. When we explored the factors driving this relationship, we discovered a significant effect of Z-scores in the late third trimester, in CHD subjects but not in Controls. The motor domain exhibited the most significant differentiation in scores, effectively distinguishing individuals with CHD from controls, showing strong associations with Z-scores of various brain regions. Our results reflect expected developmental profile of children with CHD,^75^, ^76^, ^77^, ^78^, ^79^ with the Motor domain often most affected in toddlerhood, showing a large variance between individuals.^80^ However, when projecting further into development, school-age outcomes will be shaped by other environmental factors and therefore may not share the same correlates as early 2-year outcomes. As the infants develop, their cognitive, emotional, and social abilities will become more heavily influenced by a range of interacting environmental factors, such as the broader familial and social contexts they're exposed to, therefore foetal brain structure may become a less pertinent predictor in later life.^81^, ^82^, ^83^

The foetal cortical regions that were significantly associated with neurodevelopmental outcomes in the CHD group are well-established functional areas for those developmental domains in later life. For example, the precentral gyrus contains the primary motor cortex, which is responsible for the control of voluntary motor movement,^84^ and in our study, we found that abnormal foetal precentral gyrus surface area is associated with two-year motor outcomes. Similarly, we found cognitive and language outcomes were associated almost exclusively with Z-scores in temporal lobe gyri, which is known to contain tertiary association cortex, a key substrate for higher order cognition including language and memory.^85^ This result highlights that in utero cortical areal expansion is an essential structural foundation for the optimal function of these regions in toddlerhood. Our study also reproduces observations in adolescent CHD cohorts, noting volume and thickness reductions distributed throughout parietal, medial frontoparietal, cingulate, and temporal gyri,^86^^,^^87^ suggesting that the structural brain anomalies we observe at the start of life may persist into later life.

There are several important limitations to note regarding this work. We were unable to pinpoint the specific gestational age when Z-score becomes significantly different between CHD and Control groups, or predictive of outcome. To do this accurately requires a significantly larger cohort size, so a sliding window approach could be used with equal bin sizes across the gestational age range. Another notable limitation is the demographic of this study cohort, which was not diverse or representative of the US population, containing over 90% white maternal race and ethnicity. Therefore we could not reliably assess the impact of maternal ethnicity, and the interaction with social determinants of health, despite this being an established factor in CHD aetiology.^88^^,^^89^ In a similar vein, as we continue to collect data for this study and the sample size grows, we will endeavour to assess the contribution of other clinical factors more thoroughly. Additionally, we did not assess foetal cortical thickness in this study, as the values are close to the limit of our current acquisition resolution (∼1 mm) and therefore there is a significant partial volume effect.

## Contributors

SW, CKR, and KI conceptualised the hypotheses and the methodological approach of the study. SW, CKR, and KI accessed and verified the underlying data. MRI data were collected by KI and CKR, and neurodevelopmental assessments were conducted by AS. Image processing, quality control, and extraction of neuroimaging metrics were handled by SW, SJ, and HJY, who implemented preprocessing pipelines and custom processing scripts, while SW, HAF, and KI developed and conducted the statistical analysis, including group comparisons and outcome modelling, with statistical consultation provided by HAF. All authors contributed to result interpretation, with specific insights into neurodevelopmental outcomes provided by JWN, CKR, and AS. The final manuscript was read and approved by all authors.

## Data sharing statement

Image processing and analysis code for the manuscript is available via the centralised lab Github (https://github.com/FNNDSC). Anonymised data including MRI scans, metadata and derivative files used in this study will be available upon reasonable request with investigator support (sian.wilson@childrens.harvard.edu, kiho.im@childrens.harvard.edu), after signing a data sharing and usage agreement as required by the corresponding authors' institution.

## Declaration of interests

CKR received funding from the Centres for Disease Control and the Newborn Brain Society. HAF received support from the National Institute for Health (HG-013725, NS-126792, HD-114338, HD-076258, HG-011798, HD-112697, NS-114087, EB-031170, HL-046925, EB-034757).
