# Supplementary material for: Foetal cortical expansion is associated with neurodevelopmental outcome at 2-years in congenital heart disease: a longitudinal follow-up study
Source: eBioMedicine. 2025 Mar 29;114:105679. doi: 10.1016/j.ebiom.2025.105679 (PMC11994330; doi:10.1016/j.ebiom.2025.105679)

**Supplementary Information**

**Factorial ANOVA model equations**

1. **Cross-sectional analyses**

- Independent variables:

*i =* group (CHD, Control); alternatively, (HLHS/TGA, other CHD, Control)

*j =* region (1, …, 30)

*k =*  hemisphere (L, R)

*m =* subject (1, … )

*n =* repeat scan (usually 1 only; rarely 1, 2 or 1, 2, 3)

- Terms in model:

μ = constant

α*_i_* = group effect

β*_j_* = region effect

γ*_k_* = hemisphere effect

[αβ]*_ij_* = group × region interaction

[αγ]*_ik_* = group × hemisphere interaction

[βγ]*_jk_* = region × hemispherre interaction

[αβγ]*_ijk_* = group × region × hemisphere interaction

δ*_m_* = subject effect (random; mean 0, variance τ^2^)

ε*_ijkmn_* = residual error (random; mean 0, variance σ^2^)

*(Note:* [αβ]*, etc. are compound symbols, to be read as if representing a single letter.)*

- Model equation:

y*_ijkmn_* = area Z-score for group *i*, region *j*, hemisphere *k*, subject *m*, repeat scan *n*

= sum of above terms

- Parameter estimates derived from fitted model:

ӯ*_ijk_* – ӯ*_i′jk_* = difference in mean area Z-score between groups *i* and *i′*, for region *j*, hemisphere *k*

1. **Longitudinal analysis**

- Independent variables:

*i =* group (CHD, Control); alternatively, (HLHS/TGA, other CHD, Control)

*j =* region (1, …, 30)

*k =*  hemisphere (L, R)

*m =* subject (1, … )

ΔGA*_m_* = difference in GA between earlier (<30 wk) and later scan (≥30 wk) for subject *m*

- Terms in model:

μ = constant

α*_i_* = group effect

β*_j_* = region effect

γ*_k_* = hemisphere effect

[αβ]*_ij_* = group × region interaction

[αγ]*_ik_* = group × hemisphere interaction

[βγ]*_jk_* = region × hemispherre interaction

[αβγ]*_ijk_* = group × region × hemisphere interaction

Γ × ΔGA*_m_* = effect of age/growth

δ*_m_* = subject effect (random; mean 0, variance τ^2^)

ε*_ijkm_* = residual error (random; mean 0, variance σ^2^)

*(Note:* [αβ]*, etc. are compound symbols, to be read as if representing a single letter.)*

- Model equation:

y*_ijkm_* = change in area Z-score for group *i*, region *j*, hemisphere *k*, subject *m*

= sum of above terms

- Parameter estimates derived from fitted model:

ӯ*_ijk_* – ӯ*_i′jk_* = difference in mean change of area Z-score between groups *i* and *i′*,
for region *j*, hemisphere *k*

| Covariate | Stats Test | Estimates (Mean) | Summary statistics | Result |
| --- | --- | --- | --- | --- |
| Gestational Age at Birth | T-test | CHD = 38.72  Control = 39.18 | t = -1.5615, df = 81.177, p-value = 0.1223 | Not significant |
| Maternal Age | T-test | CHD = 32.09  Control = 33.15 | t = -1.2487, df = 84.753, p-value = 0.2152 | Not significant |
| Sex | Chi-Square test | n/a | X-squared = 3.9645e-31, df = 1, p-value = 1 | Not significant |
| Maternal Education | Chi-Square test | n/a | X-squared = 5.4167, df = 4, p-value = 0.2471 | Not significant |
| Maternal Race | Chi-Square test | n/a | X-squared = 3.6753, df = 3, p-value = 0.2987 | Not significant |

**Supplementary Table 1. Statistical testing of group characteristics between CHD and Controls**

| **Supplementary Table 2. Fetal CHD Diagnoses and Subgrouping (HLHS/TGA and ‘Other’)** | | |
| --- | --- | --- |
| **Subject** | **Fetal Categorization (HLHS/TGA vs Other)** | **CHD Diagnosis** |
| Subject 1 | Other | TA with normal, related great arteries and mild PS |
| Subject 2 | HLHS_TGA | D-TGA/ IVS No outflow tract obstruction |
| Subject 3 | HLHS_TGA | HLHS (AS, MS) Severe MS and dilated LA, Restrictive atrial septum |
| Subject 4 | Other | VSD, Coarctation |
| Subject 5 | HLHS_TGA | D-TGA moderate mebranous VSD |
| Subject 6 | Other | L-TGA with large VSD, mildly hypoplastic aortic valve and arch, moderately hypoplastic RV |
| Subject 7 | Other | TOF with absent pulmonary valve, moderate RV dysfunction and dilation |
| Subject 8 | HLHS_TGA | DORV, Taussig-Bing type, subpulmonary VSD, hypoplastic aortic arch |
| Subject 9 | HLHS_TGA | D-TGA/Intact Ventricular Septum |
| Subject 10 | HLHS_TGA | DORV with D-TGA, hypoplastic aortic valve and aotic arch |
| Subject 11 | HLHS_TGA | Hypoplastic Left Heart Syndrome |
| Subject 12 | Other | Severely unbalanced Right dominant CAVC, DORV |
| Subject 13 | Other | Fetal aortic stenosis with normal Left ventricle size and mild systolic dysfunction |
| Subject 14 | Other | Fetal AS with evolving HLHS |
| Subject 15 | Other | Severe Ebstein's |
| Subject 16 | HLHS_TGA | Tricupsid atresia, D-TGA with hypoplastic aortic arch and aortic atresia |
| Subject 17 | HLHS_TGA | HLHS (AS/MS), mildy restrictive PFO |
| Subject 18 | Other | Large AP window, hemitruncus with LPA from ascending aorta |
| Subject 19 | Other | TOF/ PA |
| Subject 20 | Other | Mitral atresia, hypoplastic LV with VSD, DORV, D-TGA, PS |
| Subject 21 | Other | Fetal AS with evolving HLHS |
| Subject 22 | Other | Tricuspid Atresia, Type 1b with normally related great arteries and pulmonary stenosis |
| Subject 23 | Other | Severe valvar AS normal sized LV, mild LV systolic dysfunction PFO with BO retrograde aortic arch flow |
| Subject 24 | Other | Tetralogy of Fallot, mildy hypoplastic pulmonary valve & mpa, normal PA |
| Subject 25 | HLHS_TGA | HLHS (mA/AA) partial anomalous pulmonary veins |
| Subject 26 | Other | TOF/ PA multiple aortopulmonary collaterals, hypoplastic pulmonary arteries |
| Subject 27 | HLHS_TGA | Severe AS, moderate LV dilation, severe dysfunction, retrograde flow in transverse aortic arch |
| Subject 28 | HLHS_TGA | D-TGA, intact ventricular system |
| Subject 29 | Other | Coarctation |
| Subject 30 | Other | TOF, midly hypoplastic pulmonary valve & MPA |
| Subject 31 | HLHS_TGA | HLHS Type 3: aortic atresia and mitral stenosis |
| Subject 32 | Other | Heterotaxy, severely unbalanced right dominant CAVC, DORV, PA |
| Subject 33 | Other | TA, Type 1C |
| Subject 34 | HLHS_TGA | HLHS, intact atrial septum |
| Subject 35 | HLHS_TGA | D-TGA, large VSD, likely coarction |
| Subject 36 | HLHS_TGA | HLHS (mA/AA) and supracardiac TAPUR |
| Subject 37 | Other | Fetal AS with probable evolving HLHS |
| Subject 38 | HLHS_TGA | HLHS, Type 2: aortic atresia and mitral atresia |
| Subject 39 | Other | Fetal AS with evolving HLHS + apical LV aneurysm |
| Subject 40 | Other | Balanced CAVC |
| Subject 41 | Other | Severe Ebstein's, functional pulmonary atresia |
| Subject 42 | Other | Double Inlet Single Left Ventricle |
| Subject 43 | HLHS_TGA | HLHS MS/AA, mildly restrictive atrial septum |
| Subject 44 | Other | RV>LV size discrepancy, hypoplastic aortic arch |
| Subject 45 | HLHS_TGA | HLHS, Type 3: aortic atresia and mitral stenosis |
| Subject 46 | Other | Balanced CAVC |
| Subject 47 | Other | Critical Pulmonary Stenosis |
| Subject 48 | Other | CAVC, truncus arteriosus |
| Subject 49 | Other | Heterotaxy, single RV, PS, transposition |
| Subject 50 | HLHS_TGA | D-TGA/IVS |
| Subject 51 | Other | Tricuspid Atresia & PA |
| Subject 52 | Other | DORV |
| Subject 53 | HLHS_TGA | D-TGA, no VSD |
| Subject 54 | Other | VSD, Coarctation |
| Subject 55 | Other | DORV and Severe LV Hypoplastia |
| Subject 56 | Other | Tetralogy of Fallot |
| Subject 57 | HLHS_TGA | D-TGA |
| Subject 58 | HLHS_TGA | HLHS, Type 2: aortic atresia and mitral atresia |
| Subject 59 | Other | Coarctation |
| Subject 60 | Other | TOF |
| Subject 61 | HLHS_TGA | D-TGA |
| Subject 62 | Other | D-TGA |
| Subject 63 | Other | TOF and PA |
| Subject 64 | HLHS_TGA | D-loop intact ventricular septum |
| Subject 65 | HLHS_TGA | D-TGA/VSD/ Coarctation |
| Subject 66 | Other | TOF with PA likely biventricular circulation |
| Subject 67 | HLHS_TGA | HLHS with dominant single right ventricle |
| Subject 68 | Other | Coarctation with significant juxta-ductal shelf. Mild hypoplasia of left sided structures (mitral valve, LV, aortic valve, arch) and isthmus. |
| Subject 69 | Other | FCI for aortic stenosis and mitral regurgitation. |
| Subject 70 | Other | TOF with pulmonary stenosis with likely biventricular circulation |
| Subject 71 | HLHS_TGA | D-loop TGA with intact ventricular septum and likely biventricular circulation |
| Subject 72 | HLHS_TGA | HLHS |
| Subject 73 | Other | "pink" TOF vs isolated conoventricular septal defect with no right ventricular outflow tract abnormality. |
| Subject 74 | HLHS_TGA | D-TGA with a sub-pulmonary conoventricular septal defect and a hypoplastic aortic valve and aorta arising from the right ventricle. |
| Subject 75 | HLHS_TGA | D-TGA with intact ventricular septum |
| Subject 76 | Other | Large conoventricular septal defect with posterior deviation of conal septum.  Narrowing of the left ventricular outflow tract with mildly subnormal aortic annulus and ascending aorta diameter. |
| Subject 77 | Other | TOF with pulmonary stenosis |
| Subject 78 | HLHS_TGA | D-TGA/VSD |
| Subject 79 | HLHS_TGA | HLHS with likely mitral stenosis and aortic atresia |
| Subject 80 | Other | Double Inlet Left Ventricle, Normally related arteries (Holmes' Heart) |
| Subject 81 | HLHS_TGA | D-TGA |
| Subject 82 | Other | DORV, Moderate to Large VSD |
| Subject 83 | HLHS_TGA | Moderate to large VSD |
| Subject 84 | HLHS_TGA | D-TGA, PFO and ductus arteriosus |
| Subject 85 | Other | Double Inlet Left Ventricle |
| Subject 86 | Other | TOF, mildly hypoplastic pulmonary valve and right aortic arch. |
| Subject 87 | Other | Hypoplastic left heart variant with ventricular septal defect and interrupted aortic arch. |
| Subject 88 | Other | Valvar aortic stenosis with predominantly antegrade flow around the aortic arch. Mild mitral regurgitation. Severe left ventricular dysfunction |
| Subject 89 | HLHS_TGA | D-TGA |
| Subject 90 | HLHS_TGA | HLHS with mitral stenosis and likely aortic stenosis, severely hypoplastic ascending aorta, retrograde flow in hypoplastic transverse aortic arch. |

Abbreviations; TA: Tricuspid Atresia; D-TGA: Dextro-Transposition of the Great Arteries; IVS: Intact Ventricular Septum; HLHS: Hypoplastic Left Heart Syndrome; AS: Aortic Stenosis; MS: Mitral Stenosis; LA: Left Atrium; VSD: Ventricular Septal Defect; RV: Right Ventricle; TOF: Tetralogy of Fallot; DORV: Double Outlet Right Ventricle; PFO: Patent Foramen Ovale; CAVC: Complete Atrioventricular Canal Defect; PA: Pulmonary Atresia; LPA: Left Pulmonary Artery; LV: Left Ventricle; FCI: Fetal Cardiac Intervention; TAPUR: Total Anomalous Pulmonary Venous Return; AP Window: Aortopulmonary Window;

PS: Pulmonary Stenosis.

**Supplementary Table 3. FDR-adjusted p values for each cortical region, comparing Z-scores between CHD and Control subjects, by metric, scan number and hemisphere (L/R)**

|  | Null Hypothesis: Z-scores are the same for CHD and Control subjects | | | | | | | | | | | |
| --- | --- | --- | --- | --- | --- | --- | --- | --- | --- | --- | --- | --- |
|  | Surface Area | | | | Sulcal Depth | | | | Mean Curvature | | | |
|  | Scan 1 | | Scan 2 | | Scan 1 | | Scan 2 | | Scan 1 | | Scan 2 | |
| Label | L | R | L | R | L | R | L | R | L | R | L | R |
| caudalmiddlefrontal | 0.241 | 0.271 | 0.307 | **0.040*** | 0.159 | 0.100 | 0.501 | 0.100 | 0.211 | 0.134 | 0.425 | 0.700 |
| cingulatecortex | 0.621 | 0.332 | 0.700 | 0.271 | 0.974 | 0.912 | 0.501 | 0.912 | 0.700 | 0.332 | 0.425 | 0.549 |
| cuneus | 0.695 | 0.454 | 0.164 | 0.817 | 0.328 | 0.774 | 0.613 | 0.774 | 0.211 | 0.948 | 0.611 | 0.904 |
| frontalpole | 0.986 | 0.167 | 0.938 | 0.271 | 0.828 | 0.501 | 0.974 | 0.501 | 0.676 | 0.264 | 0.700 | 0.511 |
| fusiform | 0.834 | 0.817 | 0.254 | 0.103 | 0.050 | 0.175 | 0.752 | 0.175 | 0.264 | 0.508 | 0.828 | 0.700 |
| inferiorparietal | 0.271 | 0.220 | 0.139 | 0.268 | 0.546 | 0.660 | 0.974 | 0.660 | 0.684 | 0.479 | 0.794 | 0.425 |
| inferiortemporal | 0.938 | 0.834 | 0.560 | 0.318 | 0.058 | 0.501 | 0.501 | 0.501 | 0.332 | 0.264 | 0.589 | 0.759 |
| insula | 0.528 | 0.712 | 0.058 | 0.052 | 0.159 | 0.546 | 0.660 | 0.546 | 0.700 | 0.613 | 0.479 | 0.511 |
| isthmuscingulate | 0.753 | 0.984 | 0.621 | 0.052 | 0.681 | 0.809 | 0.752 | 0.809 | 0.700 | 0.332 | 0.828 | 0.254 |
| lateraloccipital | 0.536 | 0.182 | 0.834 | 0.182 | 0.175 | 0.667 | 0.159 | 0.667 | 0.743 | 0.425 | 0.825 | 0.700 |
| lateralorbitofrontal | 0.604 | 0.817 | **0.040*** | **0.011*** | 0.378 | 0.853 | 0.175 | 0.853 | 0.743 | 0.502 | 0.801 | 0.794 |
| lingual | 0.834 | 0.625 | 0.241 | **0.004*** | 0.501 | 0.560 | 0.889 | 0.560 | 0.112 | 0.364 | 0.700 | 0.825 |
| medialorbitofrontal | 0.182 | 0.385 | **0.002**** | 0.197 | 0.752 | 0.501 | 0.501 | 0.501 | 0.842 | 0.417 | 0.332 | 0.825 |
| middletemporal | 0.817 | 0.918 | 0.423 | 0.271 | 0.546 | 0.784 | 0.501 | 0.784 | 0.508 | 0.825 | 0.748 | 0.794 |
| paracentral | 0.621 | 0.834 | 0.438 | 0.384 | 0.895 | 0.983 | 0.866 | 0.983 | 0.649 | 0.700 | 0.700 | 0.825 |
| parahippocampal | 0.817 | 0.700 | 0.450 | 0.271 | 0.616 | 0.667 | 0.667 | 0.667 | 0.211 | 0.264 | 0.700 | 0.752 |
| parsopercularis | 0.730 | 0.139 | 0.271 | **0.011*** | 0.070 | 0.501 | 0.546 | 0.501 | 0.211 | 0.221 | 0.999 | 0.544 |
| parsorbitalis | 0.220 | 0.384 | **0.002**** | 0.799 | 0.752 | 0.974 | 0.752 | 0.974 | 0.700 | 0.700 | 0.211 | 0.511 |
| parstriangularis | 0.435 | 0.625 | 0.415 | 0.103 | 0.752 | 0.607 | 0.853 | 0.607 | 0.525 | 0.700 | 0.842 | 0.700 |
| pericalcarine | 0.753 | 0.454 | **0.034*** | 0.150 | 0.159 | 0.312 | 0.667 | 0.312 | 0.508 | 0.700 | 0.825 | 0.999 |
| postcentral | 0.839 | 0.799 | **0.0001**** | **0.034*** | 0.289 | 0.501 | 0.378 | 0.501 | 0.348 | 0.264 | 0.264 | 0.221 |
| precentral | 0.528 | 0.607 | **0.040*** | **0.034*** | 0.462 | 0.285 | 0.501 | 0.285 | 0.511 | 0.264 | 0.283 | 0.508 |
| precuneus | 0.454 | 0.368 | 0.139 | 0.476 | 0.784 | 0.752 | 0.501 | 0.752 | 0.835 | 0.510 | 0.743 | 0.700 |
| rostralmiddlefrontal | 0.543 | 0.817 | 0.454 | 0.181 | 0.501 | 0.752 | 0.974 | 0.752 | 0.283 | 0.589 | 0.682 | 0.511 |
| superiorfrontal | 0.182 | 0.454 | 0.099 | 0.384 | 0.660 | 0.892 | 0.962 | 0.892 | 0.221 | 0.382 | 0.700 | 0.975 |
| superiorparietal | 0.454 | 0.839 | 0.454 | 0.834 | 0.592 | 0.607 | 0.224 | 0.607 | 0.211 | 0.684 | 0.348 | 0.511 |
| superiortemporal | 0.825 | 0.998 | **0.036*** | 0.269 | 0.121 | 0.667 | 0.558 | 0.667 | 0.713 | 0.825 | 0.825 | 0.364 |
| supramarginal | 0.799 | 0.182 | 0.055 | 0.543 | 0.058 | 0.596 | **0.048*** | 0.596 | 0.211 | 0.264 | 0.425 | 0.425 |
| temporalpole | 0.956 | 0.239 | 0.052 | 0.271 | 0.962 | 0.912 | 0.812 | 0.912 | 0.759 | 0.752 | 0.748 | 0.842 |
| transversetemporal | 0.834 | 0.834 | 0.139 | 0.590 | 0.050 | 0.546 | 0.837 | 0.546 | 0.700 | 0.794 | 0.264 | 0.283 |

|  | CHD | | | | | | Control | | | | | |
| --- | --- | --- | --- | --- | --- | --- | --- | --- | --- | --- | --- | --- |
|  | Cognitive | | Language | | Motor | | Cognitive | | Language | | Motor | |
| Cortical Region | L | R | L | R | L | R | L | R | L | R | L | R |
| caudalmiddlefrontal | 0.142 | 0.251 | 0.306 | 0.749 | **0.009** | 0.150 | 0.983 | 0.051 | 0.493 | 0.296 | 0.982 | 0.061 |
| cingulatecortex | 0.669 | 0.634 | 0.701 | 0.892 | **0.021** | 0.112 | 0.625 | 0.996 | 0.555 | 0.875 | 0.352 | 0.433 |
| cuneus | 0.679 | 0.571 | 0.644 | 0.281 | 0.745 | 0.183 | 0.967 | 0.948 | 0.960 | 0.878 | 0.648 | 0.580 |
| frontalpole | 0.332 | 0.327 | 0.720 | 0.630 | 0.218 | **0.030** | 0.916 | 0.350 | 0.979 | 0.179 | 0.269 | 0.927 |
| fusiform | 0.696 | 0.696 | 0.560 | 0.272 | 0.955 | 0.099 | 0.849 | 0.533 | 0.970 | 0.710 | 0.451 | 0.867 |
| inferiorparietal | 0.627 | 0.584 | 0.536 | 0.558 | 0.192 | 0.210 | 0.983 | 0.712 | 0.986 | 0.959 | 0.911 | 0.289 |
| inferiortemporal | 0.145 | 0.144 | 0.096 | 0.032 | 0.060 | **0.044** | 0.535 | 0.996 | 0.933 | 0.830 | 0.541 | 0.612 |
| insula | 0.141 | 0.209 | 0.305 | 0.359 | 0.075 | 0.114 | 0.997 | 0.356 | 0.773 | 0.734 | 0.345 | 0.062 |
| isthmuscingulate | 0.187 | 0.390 | 0.102 | 0.552 | 0.212 | **0.038** | 0.983 | 0.825 | 0.979 | 0.983 | 0.803 | 0.856 |
| lateraloccipital | 0.178 | 0.078 | 0.392 | 0.297 | 0.193 | 0.165 | 0.633 | 0.168 | 0.951 | 0.239 | 0.657 | 0.610 |
| lateralorbitofrontal | 0.191 | 0.091 | 0.102 | 0.165 | 0.020 | 0.198 | 0.480 | 0.983 | 0.878 | 0.780 | 0.580 | 0.596 |
| lingual | 0.364 | 0.724 | 0.346 | 0.651 | 0.114 | 0.478 | 0.535 | 0.497 | 0.296 | 0.679 | 0.505 | 0.982 |
| medialorbitofrontal | 0.187 | 0.364 | 0.214 | 0.384 | 0.099 | **0.045** | 0.252 | 0.898 | 0.393 | 0.734 | 0.630 | 0.853 |
| middletemporal | 0.426 | 0.009 | 0.472 | 0.068 | 0.131 | 0.229 | 0.948 | 0.768 | 0.905 | 0.805 | 0.751 | 0.929 |
| paracentral | 0.594 | 0.929 | 0.878 | 0.624 | 0.371 | 0.993 | 0.224 | 0.714 | 0.585 | 0.361 | 0.306 | 0.595 |
| parahippocampal | 0.255 | 0.890 | 0.352 | 0.392 | **0.008** | 0.375 | 0.308 | 0.948 | 0.361 | 0.479 | 0.104 | 0.225 |
| parsopercularis | 0.256 | 0.364 | 0.548 | 0.412 | **0.049** | **0.008** | 0.996 | 0.124 | 0.999 | 0.585 | 0.166 | 0.684 |
| parsorbitalis | 0.208 | 0.139 | 0.417 | 0.065 | 0.814 | **0.018** | 0.121 | 0.690 | 0.296 | 0.905 | 0.403 | 0.910 |
| parstriangularis | 0.241 | 0.545 | 0.305 | 0.697 | 0.164 | 0.309 | 0.318 | 0.979 | 0.130 | 0.960 | 0.755 | 0.760 |
| pericalcarine | 0.347 | 0.341 | 0.205 | 0.168 | **0.049** | 0.382 | 0.983 | 0.422 | 0.960 | 0.709 | 0.987 | 0.793 |
| postcentral | 0.537 | 0.570 | 0.646 | 0.970 | **0.019** | **0.006** | 0.633 | 0.727 | 0.779 | 0.979 | 0.682 | 0.998 |
| precentral | 0.037 | 0.276 | 0.250 | 0.299 | **0.014** | **0.020** | 0.776 | 0.570 | 0.543 | 0.828 | 0.595 | 0.982 |
| precuneus | 0.266 | 0.485 | 0.697 | 0.996 | 0.160 | 0.100 | 0.540 | 0.765 | 0.524 | 0.979 | 0.982 | 0.982 |
| rostralmiddlefrontal | 0.303 | 0.064 | 0.624 | 0.289 | 0.084 | **0.003** | 0.996 | 0.501 | 0.983 | 0.319 | 0.664 | 0.833 |
| superiorfrontal | 0.152 | 0.117 | 0.681 | 0.445 | **0.004** | **0.014** | 0.208 | 0.948 | 0.070 | 0.855 | 0.876 | 0.932 |
| superiorparietal | 0.473 | 0.110 | 0.927 | 0.690 | **0.029** | **0.019** | 0.431 | 0.535 | 0.361 | 0.887 | 0.669 | 0.623 |
| superiortemporal | 0.017 | 0.005 | 0.242 | 0.017 | 0.160 | 0.182 | 0.489 | 0.940 | 0.665 | 0.986 | 0.760 | 0.833 |
| supramarginal | 0.187 | 0.169 | 0.756 | 0.997 | 0.081 | 0.060 | 0.349 | 0.518 | 0.593 | 0.986 | 0.682 | 0.765 |
| temporalpole | 0.658 | 0.409 | 0.624 | 0.616 | 0.437 | 0.165 | 0.242 | 0.858 | 0.960 | 0.665 | 0.803 | 0.803 |
| transversetemporal | 0.037 | 0.002 | 0.624 | 0.000 | 0.633 | 0.083 | 0.580 | 0.938 | 0.764 | 0.959 | 0.853 | 0.982 |

**Supplementary Table 4. FDR-adjusted p values for the effect of ROI Z-score in outcome in the regression model: Neurodevelopmental Outcome Score ~ ROI Z-score + Subject + Cohort + Sex + (ROI Z-score * Cohort)**

**Supplementary Table 5. FDR-adjusted p values for the effect of ROI Z-score in outcome in the regression model: Neurodevelopmental Outcome Score ~ ROI Z-score + Subject + Cohort + >30GW + (ROI Z-score*Cohort) + (ROI Z-score* >30GW)**

|  | Post-30GW GA + CHD Reference | | | | | | Post-30GW GA + Control Reference | | | | | |
| --- | --- | --- | --- | --- | --- | --- | --- | --- | --- | --- | --- | --- |
|  | Cognitive | | Language | | Motor | | Cognitive | | Language | | Motor | |
| Cortical Region | L | R | L | R | L | R | L | R | L | R | L | R |
| caudalmiddlefrontal | 0.208 | 0.404 | 0.454 | 0.950 | **0.015** | 0.295 | 0.899 | 0.055 | 0.768 | 0.140 | 0.939 | 0.101 |
| cingulatecortex | 0.873 | 0.646 | 0.853 | 0.964 | 0.056 | 0.184 | 0.629 | 0.947 | 0.935 | 0.994 | 0.589 | 0.778 |
| cuneus | 0.776 | 0.609 | 0.705 | 0.333 | 0.909 | 0.263 | 0.805 | 0.938 | 0.970 | 0.838 | 0.895 | 0.778 |
| frontalpole | 0.448 | 0.508 | 0.887 | 0.866 | 0.306 | **0.032** | 0.880 | 0.584 | 0.993 | 0.450 | 0.650 | 0.877 |
| fusiform | 0.713 | 0.643 | 0.803 | 0.459 | 0.991 | 0.146 | 0.840 | 0.666 | 0.982 | 0.970 | 0.819 | 0.939 |
| inferiorparietal | 0.621 | 0.571 | 0.695 | 0.908 | 0.309 | 0.274 | 0.923 | 0.942 | 0.982 | 0.970 | 0.962 | 0.804 |
| inferiortemporal | 0.135 | 0.166 | 0.132 | 0.066 | 0.101 | 0.062 | 0.752 | 0.938 | 0.993 | 0.966 | 0.839 | 0.932 |
| insula | 0.217 | 0.282 | 0.543 | 0.705 | 0.110 | 0.250 | 0.931 | 0.573 | 0.970 | 0.584 | 0.851 | 0.212 |
| isthmuscingulate | 0.208 | 0.386 | 0.113 | 0.542 | 0.309 | **0.041** | 0.923 | 0.702 | 0.970 | 0.970 | 0.939 | 0.939 |
| lateraloccipital | 0.255 | 0.069 | 0.454 | 0.376 | 0.274 | 0.225 | 0.670 | 0.174 | 0.970 | 0.289 | 0.850 | 0.631 |
| lateralorbitofrontal | 0.208 | 0.059 | 0.230 | 0.212 | 0.061 | 0.320 | 0.670 | 0.880 | 0.762 | 0.808 | 0.548 | 0.882 |
| lingual | 0.507 | 0.776 | 0.454 | 0.936 | 0.191 | 0.759 | 0.678 | 0.932 | 0.614 | 0.970 | 0.850 | 0.939 |
| medialorbitofrontal | 0.162 | 0.508 | 0.408 | 0.662 | 0.158 | 0.085 | 0.544 | 0.805 | 0.494 | 0.970 | 0.924 | 0.924 |
| middletemporal | 0.507 | **0.016** | 0.629 | 0.117 | 0.208 | 0.445 | 0.747 | 0.666 | 0.970 | 0.910 | 0.924 | 0.939 |
| paracentral | 0.966 | 0.953 | 0.773 | 0.781 | 0.648 | 0.956 | 0.081 | 0.949 | 0.403 | 0.578 | 0.434 | 0.858 |
| parahippocampal | 0.305 | 0.930 | 0.537 | 0.530 | **0.015** | 0.589 | 0.328 | 0.826 | 0.551 | 0.651 | 0.351 | 0.339 |
| parsopercularis | 0.208 | 0.457 | 0.586 | 0.629 | 0.052 | **0.013** | 0.573 | 0.235 | 0.542 | 0.935 | 0.939 | 0.825 |
| parsorbitalis | 0.189 | 0.165 | 0.806 | 0.099 | 0.909 | **0.047** | 0.207 | 0.923 | 0.673 | 0.990 | 0.764 | 0.888 |
| parstriangularis | 0.465 | 0.619 | 0.630 | 0.853 | 0.320 | 0.405 | 0.625 | 0.923 | 0.448 | 0.994 | 0.939 | 0.961 |
| pericalcarine | 0.509 | 0.514 | 0.369 | 0.336 | 0.091 | 0.592 | 0.835 | 0.938 | 0.993 | 0.982 | 0.966 | 0.924 |
| postcentral | 0.546 | 0.619 | 0.736 | 0.950 | 0.053 | **0.018** | 0.653 | 0.652 | 0.970 | 0.970 | 0.903 | 0.939 |
| precentral | **0.039** | 0.281 | 0.369 | 0.530 | **0.031** | **0.050** | 0.923 | 0.805 | 0.791 | 0.993 | 0.819 | 0.939 |
| precuneus | 0.303 | 0.573 | 0.908 | 0.994 | 0.266 | 0.120 | 0.678 | 0.868 | 0.795 | 0.994 | 0.939 | 0.903 |
| rostralmiddlefrontal | 0.508 | 0.076 | 0.908 | 0.431 | 0.163 | **0.008** | 0.844 | 0.578 | 0.970 | 0.522 | 0.882 | 0.939 |
| superiorfrontal | 0.143 | 0.143 | 0.773 | 0.600 | **0.006** | **0.032** | 0.187 | 0.847 | 0.086 | 0.970 | 0.888 | 0.939 |
| superiorparietal | 0.618 | 0.217 | 0.964 | 0.821 | 0.082 | **0.015** | 0.528 | 0.629 | 0.354 | 0.994 | 0.816 | 0.947 |
| superiortemporal | **0.029** | **0.018** | 0.444 | **0.050** | 0.253 | 0.340 | 0.805 | 0.670 | 0.970 | 0.970 | 0.873 | 0.995 |
| supramarginal | 0.168 | 0.285 | 0.908 | 0.950 | 0.132 | 0.127 | 0.332 | 0.688 | 0.722 | 0.970 | 0.858 | 0.877 |
| temporalpole | 0.618 | 0.508 | 0.812 | 0.869 | 0.759 | 0.257 | 0.464 | 0.947 | 0.970 | 0.808 | 0.838 | 0.961 |
| transversetemporal | 0.083 | **0.010** | 0.919 | **0.001** | 0.788 | 0.171 | 0.931 | 0.753 | 0.970 | 0.970 | 0.912 | 0.932 |

**Supplementary Figure 1. Flowchart showing number of scans excluded at each stage of processing, and the quality control stages (QC)**


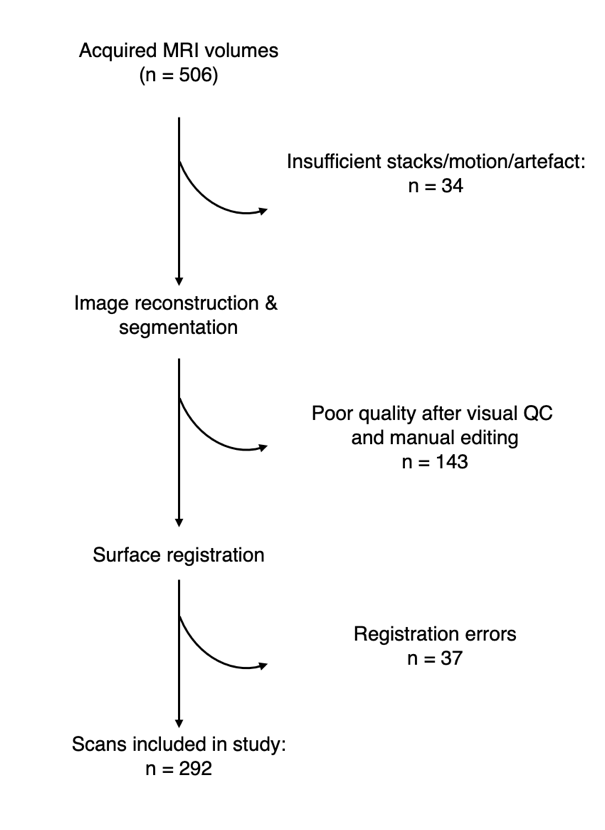

Supplement: Supplementary Materials [file mmc1.docx]
